# OligoN‐Design: A Simple and Versatile Tool to Design Specific Probes and Primers From Large Heterogeneous Datasets

**DOI:** 10.1111/1755-0998.70140

**Published:** 2026-04-12

**Authors:** Miguel M. Sandin, Marie Walde, Nicolas Henry, Irene Forn, Nathalie Simon, Cédric Berney, Ramon Massana, Daniel J. Richter

**Affiliations:** ^1^ Institut de Biologia Evolutiva (CSIC‐Universitat Pompeu Fabra) Barcelona Spain; ^2^ Sorbonne Université, CNRS, AD2M, UMR 7144, Station Biologique De Roscoff Roscoff France; ^3^ Sorbonne Université, CNRS, FR2424, RCC, Station Biologique de Roscoff Roscoff France; ^4^ CNRS, Sorbonne Université, FR2424, ABiMS, Station Biologique de Roscoff Roscoff France; ^5^ Institut de Ciències del Mar (CSIC) Barcelona Catalonia Spain

**Keywords:** bioinformatics, fluorescence in situ hybridization, oligonucleotide design, polymerase chain reaction, ribosomal DNA

## Abstract

High‐throughput environmental DNA sequencing has ushered ecological and evolutionary studies into the big data era. With thousands to millions of DNA sequences, designing taxon‐specific oligonucleotides is a current bottleneck of molecular studies that rely on primers for Polymerase Chain Reactions (PCRs) or probes for Fluorescence in situ Hybridization (FISH). No software currently exists to design specific oligonucleotides starting from a custom set of sequences. Existing tools rely on specific databases, alignments or phylogenetic trees, or cannot accommodate increasingly large molecular environmental datasets. Here we present oligoN‐design, a versatile tool to design oligonucleotides specific to a set of target sequences while minimizing predicted binding to non‐target sequences. OligoN‐design is simple, reproducible and adaptable to high‐throughput sequencing data analyses. It requires only two fasta files as input, one containing target taxa and the other containing non‐target taxa. Using standard bioinformatic formats, it integrates easily with other tools such as BLAST, VSEARCH or MAFFT. OligoN‐design allows a range of strategies that we present in detail, from an unsupervised end‐to‐end usage all the way to a detailed and thorough expert usage. Starting with large, comprehensive ribosomal databases that are widely used by the community (i.e., PR2, SILVA) and the unsupervised function, we were able to replicate known taxa‐specific oligonucleotides in under 30 min and up to 6 GB of RAM on a personal laptop. OligoN‐design, available at github.com/MiguelMSandin/oligoN‐design under GNU General Public Licence version 3.0, is easily installed via bioconda bioconda.github.io/recipes/oligon‐design/README.html.

## 1 Introduction

Environmental DNA sequencing has unveiled an enormous amount of previously unseen diversity, from uncultured taxa within known lineages to a panoply of novel groups at different taxonomic scales, from prokaryotes to eukaryotes. Environmental sequence datasets produced today include short hypervariable regions from the universal Small SubUnit of the ribosomal DNA (SSU rDNA: 16S rDNA in prokaryotes and 18S rDNA in eukaryotes; e.g., Pernice et al. 2015; Alberti et al. 2017), but also full length rDNA from metagenomes and metatranscriptomes (e.g., Krabberød et al. 2025) or direct long‐read sequencing (e.g., Jamy et al. 2019). In addition, restricted taxonomic groups have been studied using other molecular markers, for example the Internal Transcribed Spacer (ITS) of the ribosomal operon in fungi (Abarenkov et al. 2024), the mitochondrial Cytochrome Oxidase c subunit I (COI) in animals or macroalgae (González‐Miguéns et al. 2025) or the 12S and 16S rDNA of the mitochondrial gene in animals (Leray et al. 2022). The combination of different approaches and increasingly large environmental datasets has enabled a more detailed characterization of both novel environmental groups and well‐defined taxonomic groups. Many of these groups can only be studied through culture‐independent molecular approaches (e.g., Massana et al. 2014; Sandin, et al. 2025) and many others escape universal primers (Vaulot et al. 2022). The design of specific oligonucleotide primers or probes (short nucleic acid sequences of ~16–20 bases complementary to RNA or DNA signature regions of the target) could therefore accelerate the study of their biodiversity and biogeography.

Given the genetic heterogeneity of environmental datasets, and the large number of sequences they can include (from 100 s to 100,000 s), oligonucleotide design is a current bottleneck for molecular‐based ecological and evolutionary analysis of specific lineages. Both the Polymerase Chain Reaction (PCR) and Fluorescence in situ Hybridization (FISH) rely on specific complementary oligonucleotides to detect sequences of interest, and despite the fact that they are routine approaches, oligonucleotide design still requires manual and tedious work. Several software packages have been developed to aid in the design of specific oligonucleotides, such as ARB (Ludwig et al. 2004), primer3 (Untergasser et al. 2012), DECIPHER (Wright 2016), PrimerMiner (Elbrecht and Leese 2017), oli2go (Hendling et al. 2018) or OligoMiner (Beliveau et al. 2018), among others (Hendling and Barišić 2019). However, these tools require at least one input that is not easily available for large environmental datasets: sequence alignments, phylogenetic trees, specific databases and/or genomes. In addition, their learning curves might be steep, with many different parameters that might be cryptic for the non‐expert; they may allow for only a single input target sequence, they may poorly accommodate increasingly large and genetically heterogeneous molecular environmental datasets or they are no longer available.

To overcome these limitations, we introduce oligoN‐design, a simple open‐source tool to design specific oligonucleotides to be used as primers for PCR, probes for FISH or any other application. OligoN‐design is simple, versatile and reproducible since the code and environment can be saved and rerun, accommodates large environmental, high‐throughput and heterogeneous datasets and does not require phylogenetic trees, alignments or specific databases as input. It uses common formats in bioinformatics, such as FASTA files and tab‐delimited tables, so it can also be directly integrated with different tools such as VSEARCH (Rognes et al. 2016), BLAST (Camacho et al. 2009) or MAFFT (Katoh and Standley 2013), among others. OligoN‐design only requires two fasta files as input, one containing the sequences of the target set of taxa and one containing the sequences of the excluding groups (i.e., all non‐target sequences). OligoN‐design is sufficiently versatile to be accessible to users of different bioinformatic expertise: it allows the novice user to run an unsupervised oligonucleotide search, while at the same time, an expert user might take advantage of advanced functions to inspect homology in specific regions of the target sequence. Earlier versions of the oligoN‐design tool have been successfully used for designing both FISH probes (e.g., Sørensen et al. 2023) and PCR primers (e.g., Pardasani et al. 2025), demonstrating its practical use. Here, we present the rationale and structure of oligoN‐design, show typical use cases and discuss its advantages and limitations.

## 2 The oligoN‐Design Tool

OligoN‐design is a collection of functions to ease the tedious work of oligonucleotide design. Probe and primer oligonucleotides are typically designed to be as specific as possible to the group of interest (the target) and potential pitfalls in their design include false‐positive matches to non‐target groups (e.g., Vaulot et al. 2022), low specificity of the oligonucleotide in suboptimal annealing conditions (e.g., Dieffenbach et al. 1993), self‐binding of the oligonucleotide (hairpins, self‐dimers; e.g., Nazarenko et al. 2002) or poor accessibility of the targeted region within the secondary structure of the targeted molecule (e.g., Behrens et al. 2003), among others (see e.g., Hendling and Barišić 2019 for a review on in silico challenges and approaches).

**FIGURE 1:**
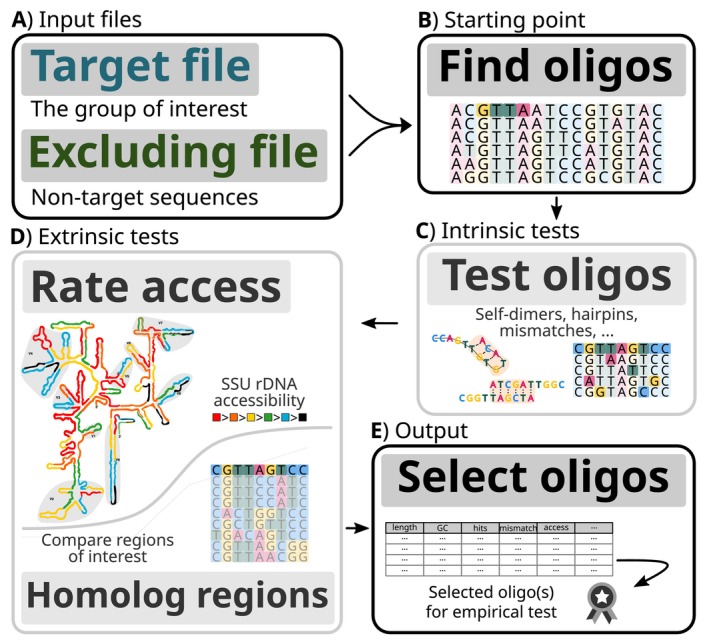
Schematic representation of the oligoN‐design tool and workflow. Black boxes represent mandatory steps, grey boxes represent optional steps. Briefly, (A) from two input files containing the target sequences and the non‐target sequences, (B) the oligoN‐design tool starts by finding all potential oligonucleotides. These oligonucleotides can then be tested for (C) intrinsic properties such as self‐dimers, hairpins or mismatches against the non‐target sequences and/or (D) extrinsic properties such as the accessibility in the gene's secondary structure or compared against homologous regions in the non‐target sequences. Lastly, (E) the best suitable oligonucleotides are listed based on all combined properties for further empirical test in the laboratory. For more details, please see Figure S1.

To mitigate all of these issues, oligoN‐design provides a series of individual steps linked together in a workflow. First, oligoN‐design takes a *target* fasta file containing target sequences and searches for regions conserved in these sequences but absent in the non‐target sequences contained in an *excluding* fasta file (Figure 1). Once specific regions have been found, these can be further studied with dedicated oligoN‐design functions in order to predict mismatches, hairpins, self‐dimers and/or the region accessibility based on the secondary structure, among other parameters. They can also be directly inspected against homologous regions within the *excluding* file for a more detailed and thorough examination of the position of the mismatches and their identity. Altogether, criteria based on these parameters are used to select candidate oligonucleotides for further empirical tests in the laboratory.

Below is a brief description of the logic of oligoN‐design, its main functions and how each function can be integrated to facilitate the design of specific oligonucleotides. For a detailed and complete description of all functions and fully documented replicable workflows please refer to the documentation manual and for a graphical detailed overview of the tool see Figure S1. The oligoN‐design tool is mostly written in Python 3, relying on dependencies that are commonly installed in bioinformatics compute clusters, such as MAFFT (Katoh and Standley 2013) for aligning sequences, HMMER (hmmer.org/) to search homologous regions and agrep (Wu and Manber 1992) for approximate matching or common python libraries such as Biopython (Cock et al. 2009), pandas (The Pandas Development Team 2024) and Matplotlib (Hunter 2007). The tool is available at github.com/MiguelMSandin/oligoN‐design (and the long‐term repository Zenodo with doi: 10.5281/zenodo.7473194) under the GNU General Public Licence version 3.0 and can be easily installed from Bioconda (bioconda.github.io/recipes/oligon‐design/README.html) together with all its dependencies.

### 2.1 A Collection of Versatile Functions

Designing oligonucleotides is a diverse task that is approached from many different angles depending on the user's experience. A novice user with limited experience in bioinformatic analysis and oligonucleotide design might simply be interested in the top 4 best‐scoring oligonucleotides. However, a more experienced user might be interested in recalibrating search parameters, interacting with intermediate files, or applying different approaches or functions from other packages. The thorough user might even be interested not only in the number of mismatches, but in the position and the identity of the mismatched hit. And an expert user might be interested in inspecting the alignment manually. However, given the sizes of environmental datasets, the visual inspection of an alignment could be challenging if not meaningless. In that case, the expert user might be interested in reducing the dataset to only the relevant homologous region. Altogether, from an unsupervised run to expert usage, oligoN‐design offers different functions to accommodate the user's preferred workflow.

**FIGURE 2:**
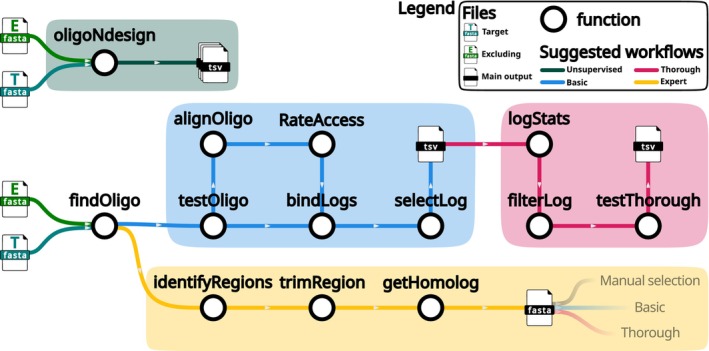
Different workflows suggested in this manuscript and detailed in the text. Briefly, the unsupervised workflow is the automatic implementation of the basic workflow, in which oligonucleotides are found and tested for mismatches and their accessibility is rated in the tertiary structure of the rDNA to select the N (by default *N* = 4) best scoring oligonucleotides. The thorough workflow starts from a tab delimited table (either resulting directly from the oligonucleotides found or from the final output of the basic workflow), filters only oligonucleotides of interest based on global statistics defined by the user, and applies a thorough test to explore the position and identity of the mismatches. Lastly, the expert workflow identifies potential regions of interest (as opposed to given oligonucleotides of defined lengths) and extracts homologous regions to later apply a manual selection of the oligonucleotides, or instead to use the output as a starting target and excluding files as input to a new run of the basic and/or thorough workflow.

Here we suggest 4 different workflows (Figure 2) to introduce the main functions and to showcase the versatility and integration of the oligoN‐design tool. These workflows go from an end‐to‐end unsupervised usage of the oligoN‐design tool to an expert usage. A detailed and hands‐on report of each workflow can be found in the documentation manual and replicable example scripts can be found in the directory ‘pipelines’ from the github repository.

### 2.2 Unsupervised Run

The simplest usage of the oligoN‐design tool is running the unsupervised wrapper function *oligoNdesign* (Figure 2, dark green workflow; corresponding to the functions of the basic workflow described below, with default options). Briefly, this function will search specific oligonucleotides to the *target* fasta file that are not in the *excluding* fasta file, test them, rate their accessibility in the SSU (if applicable) and select the 4 best scoring oligonucleotides. For specific details see the following section ‘2.3 Basic workflow’. For practical examples of this workflow, see ‘4. Recommendations based on a qualitative performance’ further down.

### 2.3 Basic Workflow

**FIGURE 3:**
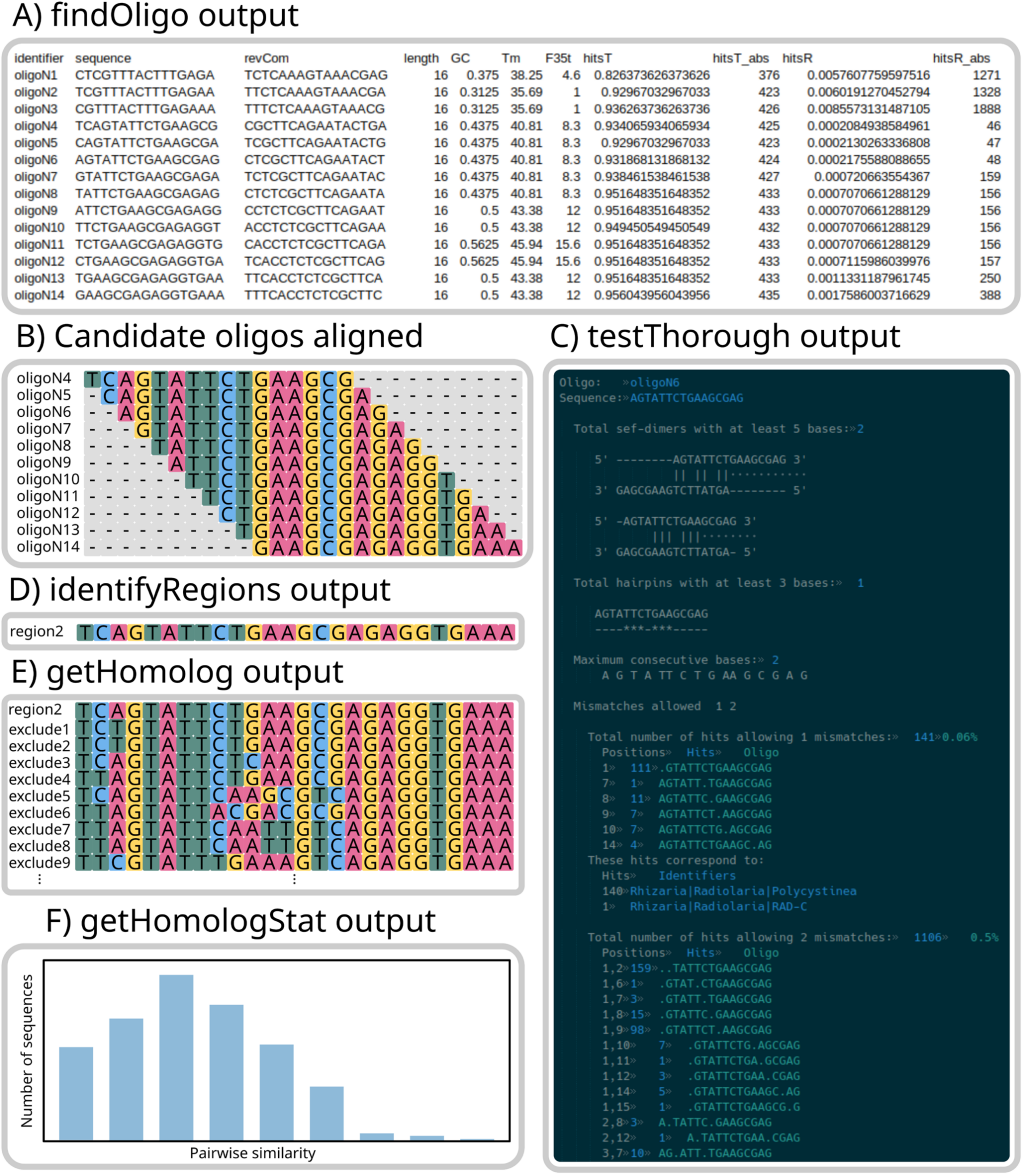
Example outputs of different functions of oligoN‐design: (A) a screenshot of a tab delimited table output from *findOligo*; (B) a schematic representation of the candidate oligonucleotides obtained from *findOligo* as sliding windows of length *k* from a region of length > *k*; (C) a screenshot of the *testThorough* output; (D) a fasta file output from *identifyRegions*; (E) a fasta file output from *getHomolog*; (F) a plot prompted from *getHomologStats*.

The basic workflow is the supervised version of the *oligoNdesign* function. Here, the user can interact with all intermediate files, arguments and options, which allows tuning and optimizing user‐defined thresholds according to the data and needs. The main starting point is the function *findOligo* (Figure 2, blue workflow). By default it searches for specific regions of length 18 and 20 bases that are present in at least 80% of the sequences from the *target* file and in less than 1% of the sequences in the excluding file. These thresholds were chosen based on empirical evidence of the most common specific oligonucleotides properties but can be adapted by the user based on their experimental requirements (as for any other threshold within oligoN‐design). As a result, a tab‐delimited table is exported, containing the identified candidate oligonucleotides and several values such as the GC content and the number and proportion of hits in the *target* and *excluding* files (Figure 3A).

The candidate oligonucleotides found with the *findOligo* function (Figure 3B) are subsequently tested for mismatches to optimize specificity and minimize false‐positive hybridizations, as follows: the function *testOligo* takes advantage of the ‘agrep’ program (Wu and Manber 1992) for fast and approximate matching to test for hits to the *excluding* file allowing mismatches like insertions and deletions. For datasets focusing on the SSU rDNA, candidate oligonucleotides can also be tested for their accessibility in the secondary structure of the SSU rDNA after the empirical results from (Behrens et al. 2003). Briefly, *alignOligo* aligns the candidate oligonucleotides to the SSU template and *rateAccess* extracts the empirical accessibility. The user can also benefit from the function *rateAccess* for other genes if a custom accessibility table is provided (see documentation or help function for further details).

Selecting potential oligonucleotides from all candidate oligonucleotides will depend on the user's preferences, experience and most importantly, the question of the study. By default, all search functions from oligoN‐design will output a tab‐delimited table with values and parameters specific to the function used (see documentation manual for details about the specific output of each function). Different tables can be merged with the function *bindLogs* and then the user can select the best oligonucleotides based on the resulting values (e.g., high specificity, high GC content, low hits to the excluding file allowing mismatches, highly accessible, etc.). Additionally, the best scoring oligonucleotides can be ranked with the function *SelectLog* and thus the novice user can select the best oligonucleotides without relying on subjective criteria.

### 2.4 Thorough Design

More thorough tests can also be applied with the function *testThorough* (Figure 2, magenta workflow, see Figure 3C for an example output), including evaluating the risks of hairpin and homodimer formations, maximum consecutive bases, mismatches, their exact position in the oligonucleotide and the identity of the mismatched sequence. The desired position of mismatches depends on whether the oligonucleotide will be used as a hybridization probe (where central mismatches are favoured) or as a PCR primer (where the most important mismatches are close to the 3′ end where DNA polymerization starts). Since these analyses are relatively computationally expensive, we suggest that the user filters the searched oligonucleotides and provides a small input file, for example providing the best scoring oligonucleotides from the basic workflow or from the *findOligo*'s output. This will yield a more efficient computation by only performing the thorough tests on oligonucleotides that will most likely be kept for empirical laboratory experiments. To do so, the user can explore the obtained parameters with the function *logStats*, that will produce summary statistics (i.e., percentiles, mean) from all different properties and manually filter oligonucleotides of interest with the function *filterLog*.

### 2.5 Expert Design

Lastly, and closest to the traditional approach to designing oligonucleotides, is the ‘simple’ examination of the alignment. However, given the large number of available sequences, this approach is becoming increasingly impractical and a considerable amount of experience is needed. To reduce the size of the alignment to be examined, the oligoN‐design tool can be used to identify specific regions of interest from the *target* file and search for homologous regions in the *excluding* file (Figure 2, yellow workflow).

Candidate oligonucleotides are frequently sliding windows of a given size from specific regions (Figure 3B) that can be grouped and identified with the *identifyRegions* function (Figure 3D). Then, homologous regions from the *excluding* file are retrieved with the function *getHomolog* (Figure 3E) using an HMM profile from the HMMER package (hmmer.org/). The resulting fasta file can be analysed with *getHomologStats* to estimate the pairwise similarity of all sequences of a fasta file against the first sequence in the file to produce a histogram with the similarities (Figure 3F). These regions can also be used as a starting point to apply a basic or a thorough workflow on highly curated *target* and *excluding* files, thus continuing with an automated and replicable pipeline. This approach speeds up computational analysis for testing mismatches, while at the same time allowing a greater variety of oligonucleotide lengths and visually inspecting homologous regions (e.g., checking for variable positions in the *target* file, mismatches against the *excluding* file, the position of the mismatches, etc.).

### 2.6 Other Complementary Aspects and Functions

Even though alignments are not strictly required for oligoN‐design, we recommend that advanced users align, specifically, the *target* file to properly understand both the file and the group of interest (see ‘Good practices’ on the documentation manual for further details). Such a practice will help the advanced user fine‐tune parameters related to the specificity and probably discard or focus on specific regions of the gene that have a low or a high coverage, respectively. However, large alignments are sometimes difficult to visualize; the function *alignmentConsensus* creates a consensus sequence to quickly see highly variable or conserved regions within the group of interest. And in the case that computing resources do not allow aligning a given file, the function *breakFasta* extracts all unique k‐mers of length *k* and their presence abundance in the input file (including an option to plot a scatter plot with such abundances) to help the user select different oligonucleotide lengths. For further details about these and other auxiliary and helper functions, please see the ‘Quick overview of the different functions and their usage’ on the documentation manual (page 6).

All these functions allow the user to assemble a fully reproducible and end‐to‐end workflow, while at the same time letting an expert user visually inspect regions of interest. This versatility allows the integration of other complementary and already available tools for oligonucleotide design, such as primer3 (Untergasser et al. 2012), a widely used programme that focuses on thermodynamic properties of the oligonucleotide.

## 3 Novelty and Complementary Aspects

OligoN‐design complements existing software and helps the user streamline the process of oligonucleotide design in the age of environmental omics. OligoN‐design focuses mostly on specificity, accommodating large and heterogeneous environmental datasets.

Of existing oligonucleotide design software, probably the most widely used for probe and primer design in ecological and evolutionary studies is the ARB project (Ludwig et al. 2004) integrated with the SILVA database of the SSU rDNA (Quast et al. 2013). Briefly, this interactive tool allows the user to navigate a phylogenetic tree (and a very large alignment) and to select the taxa of interest in the tree. Signature regions in the target sequences and mismatches in the non‐target sequences can be visualized in alignments as well as in models of secondary gene structure. Although ARB has proven very successful for oligonucleotide design, it has a steep learning curve and many dependencies and requires a previous alignment and a phylogenetic tree, making it difficult to accommodate custom databases. Primer3 (Untergasser et al. 2012), another widely used tool for oligonucleotide design (mostly in biomedical studies), incorporates accurate thermodynamic models to improve the prediction of the melting temperature and therefore minimize empirical optimization in the laboratory. However, Primer3 only accepts one input sequence. Lastly, the R packages DECIPHER (Wright et al. 2014; Wright 2016) and PrimerMiner (Elbrecht and Leese 2017) allow designing target‐specific oligonucleotides from alignments and creating databases in compressed native formats, yet permit a relatively low heterogeneity within the target sequences. In this landscape of oligonucleotide design software, the oligoN‐design tool introduces a simple and versatile command‐line approach designed to accommodate large, heterogeneous and custom environmental datasets into bioinformatic analysis.

## 4 Recommendations Based on a Qualitative Performance

We checked the qualitative performance of oligoN‐design by applying a posteriori search for commonly used oligonucleotides on large and comprehensive empirical databases including the EukRibo (v2; Berney et al. 2022), SILVA (v138.2.NR99; Quast et al. 2013) and the Protist Ribosomal Reference (PR2 v5.0.0; Guillou et al. 2013) databases of SSU sequences. More precisely, we searched for randomly‐selected general and specific FISH probes described as ‘very good’ in the Table S1 from (Piwosz et al. 2021), using the unsupervised function oligoNdesign. The different scope of the databases yielded very different results when retrieving and identifying already known oligonucleotides (or FISH probes in this example; Table 1). While all three databases are meant to taxonomically assign reads from High Throughput Sequencing, they all differ in, for example, their quality controls or naming strategies, and therefore the input files must be independently curated. Below, a brief description of the yielded probes, the curation strategies and further recommendations to the user.

**TABLE 1:** Qualitative performance of an unsupervised search (with the function *oligoNdesign*) for already published oligonucleotide probes using different databases (EukRibo, SILVA and PR2) showing the number of sequences in the *target* file (N), the average genetic diversity (measured as the average similarity identity by applying the ‘‐‐allpairs_global’ from VSEARCH) of the *target* file (S) and whether the given probe was found (✔) or not (✘) (F).

Target	Target oligonucleotide	EukRibo			PR2			SILVA		
		N	S	F	N	S	F	N	S	F
Eukaryota	GGGCATCACAGACCTG (EUK1209R)/ACCAGACTTGCCCTCC (EUK516)	NA	NA	NA	212,926	68.6 sd: 13.6	✘	58,941	72.3 sd: 8.7	✔
Haptophyta	GGAATACGAGTGCCCCTGAC (PRYM02)	258	91.9 sd: 3.8	✔	1201	92.24 sd: 5.7	✔	228	90.6 sd: 5.1	✔
MAST 1B	AACGCAAGTCTCCCCGCG (NS1B)	3	98.7 sd: 0.7	✔	1201	92.2 sd: 5.7	✔	228	90.6 sd: 5.1	✔
MAST‐12C	TACAGTGCCAATGGAGAC (MAST‐12Nor2)	4	96.3 sd: 0.6	✔	17	96.0 sd: 2.5	✔	11	92.0 sd: 4.6	✔
Pelagophyceae	ACGTCCTTGTTCGACGCT (PELA01)	74	95.4 sd: 4.1	✔	270	96.4 sd: 5.5	✔	38	92.3 sd: 6.3	✔
*Minorisa minuta*	TACTTAGCTCTCAGAACC (CRN02)	1	NA	✔	5	99.2 sd: 0.7	✔	2	98.5	✔
MAST‐7	TCATTACCATAGTACGCA (NS7)	9	95.0 sd: 1.9	✘	235	97.2 sd: 2.6	✔	27	95.3 sd: 2.1	✔
Cercozoa Novel Clade 2	AGAACCCGTAGTCCTATA (Cerc_Bal02A)	10	91.5 sd: 2.4	✔	55	90.5 sd: 5.5	✘*	15	91.7 sd: 2.3	✘*
Cercozoa Novel Clade 2	TTCGACGTATAAGGGTGC (Cerc_Bal02B)	4	94.8 sd: 2.6	✘*	9	94.6 sd: 3.3	✘*	5	95.1 sd: 2.3	✔
Total number of sequences		46,346			221,085			510,495		
Average sequence length (and standard deviation) in bases		1980 (sd: 10605)			1411 (sd: 460)			1464 (sd: 160)		

*Note:* ‘NA’ represents ‘Not Applicable’ (i.e., lack of the target or excluding group in the given data), ‘sd’ represents ‘standard deviation’ of the given average and ‘*’ after ‘✘’ means the probe was found after thorough manual curation of the target file. Note that different lengths have been applied to accommodate the target oligonucleotide (with the option ‘‐l’). Additionally, the accessibility was not tested when targeting Eukaryota (with the option ‘‐a’) due to the large amount of sequences in the *target* file hindering alignment of the sequences.

General eukaryotic probes (such as EUK1209R; Giovannoni et al. 1988; or EUK516; Amann et al. 1990) were only retrieved in the SILVA database. It is known that general probes do not cover all targeted diversity (Vaulot et al. 2022), and these general probes were not found in PR2 mostly due to the extensive coverage of eukaryotic sequences (and small proportional coverage of prokaryotes) compared to the SILVA database. Both EUK1209R and EUK516 hit ~60% of eukaryotic sequences in PR2. For such reasons, no general eukaryotic oligonucleotide was identified using the PR2 database.

When it comes to specific oligonucleotides, the success rate was overall higher. For example, the probes PRYM02 specific to Haptophyta (Lange et al. 1996), NS1B specific to MAST‐1B (Massana et al. 2006), MAST‐12Nor2 specific to MAST‐12C (Kolodziej and Stoeck 2007) and PELA01 specific to Pelagophyceae (Simon et al. 2000) were identified in all 3 databases. The probe CRN02 specific to *Minorisa minuta* (Campo et al. 2013) was also found in all three databases; however, these databases had to be curated independently and in detail, since the three databases named *Minorisa minuta* sequences differently. While EukRibo and PR2 had only 1 and 5 sequences identified as ‘*Minorisa minuta*’, respectively, SILVA had no sequences identified at the species level (but as ‘Minorisa sp.’) and therefore specific sequences had to be extracted by matching accession numbers.

The probe NS7 specific to MAST‐7 (Giner et al. 2016) was only found in PR2 and SILVA, because it has a low specificity to clades MAST‐7C and MAST‐7D (which has 1 mismatch difference) and EukRibo contains only non‐redundant diversity (i.e., the 163 sequences assigned to MAST‐7A had a similarity of 99.1% (sd:0.7%) between them). Such selection of sequences reduces the 235 sequences assigned to MAST‐7 from PR2 to just 9 in EukRibo, and thus the probe NS7 matches 66.6% of sequences in EukRibo, 87.7% in PR2 and 81.5% in SILVA. Lastly, this subclade diversity was the reason that the probes Cerc_Bal02A and Cerc_Bal02B, specific to different subclades within Cercozoa‐Novel‐Clade‐2 (Piwosz and Pernthaler 2011), were found in only one database each. Both Cerc_Bal02A and Cerc_Bal02B probes are specific to different sets of non‐monophyletic diversity comprising around 37% and 11% of Cercozoa‐Novel‐Clade‐2 sequences. Therefore, to retrieve such probes a manual selection of specific sequences according to the original scientific question is needed, in which case the probe is found.

We have additionally tested the qualitative performance of oligoN‐design on different marker genes, such as the mitochondrial cytochrome oxidase subunit I gene (COI, using the eKOI database; González‐Miguéns et al. 2025), the 12S and 16S mitochondrial rDNA genes (using the MIDORI2 database; Leray et al. 2022) and the Internal Transcribed Spacer (ITS) of the rDNA operon (using the UNITE database; Abarenkov et al. 2024). We limited our qualitative test to the default files on randomly selected groups of different numbers of sequences and an unsupervised search (ignoring the accessibility rating with the option ‘‐a’ and allowing a wide diversity of oligonucleotide lengths: ‘‐l 30 27 24 22‐18 12‐6’). In all tested databases we found resulting oligonucleotides for most of the studied cases (see Table S1 for details), yet the higher mutation rates of these genes compared to 18S rDNA generate high variability among target sequences, resulting in abnormally small oligonucleotides (< 15 base pairs in most cases). The detailed curation of the input files (mostly by restricting the target file to small, homogeneous lineages) may solve this issue, as it has been the case for the 18S rDNA (Table 1).

Based on these observations and further usage of the oligoN‐design tools, we have included ‘Common problems and misconceptions’ and ‘Good practices’ sections in the manual documentation, where we have explained in detail recommendations and we will continuously update in the future. Briefly, we strongly recommend that, regardless of the software chosen for oligonucleotide design, to begin with a literature research and to further understand the diversity of your group of interest (by for example building a phylogenetic tree). When using oligoN‐design, both the target group and input files should be properly examined and should contain the diversity that best matches the given scientific question. The oligoN‐design tool will facilitate the design of specific oligonucleotides, although it is the user's responsibility to provide a properly curated *target* and *excluding* files for the accurate detection of specific oligonucleotides.

Oligonucleotide design is a tedious job that requires a final empirical test for its completion. Therefore, bioinformatic pipelines will only provide theoretical candidate regions that have to be tested in the laboratory. Additional post hoc resources can contribute to the in silico selection of experimental conditions for the selected oligonucleotides, such as oligoCalc and/or properties calculator. These resources are capable of estimating very accurately theoretical melting temperature and/or formamide concentration, and therefore increase the success during empirical tests in the wet lab.

## 5 Quantitative Performance

**FIGURE 4:**
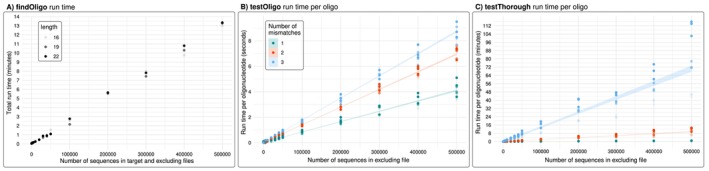
Run time of the three most computationally demanding functions using randomly‐generated fasta files of different numbers of sequences (see text for further details) and comparing oligonucleotides of different lengths (16, 19 and 22 bases). (A) Total run time in minutes of the function *findOligo* considering equal sizes for the target and excluding files. However, note that in most practical cases the target file will be significantly smaller than the excluding file, speeding up computation time. (B) Run time per oligonucleotide in seconds of the function *testOligo* considering 1, 2 and 3 mismatches for different oligonucleotide lengths. (C) Run time per oligonucleotide in minutes of the function *testThrough* considering 1, 2 and 3 mismatches for different oligonucleotide lengths.

The performance of the most computationally demanding functions was measured in time (Figure 4) and in memory usage against randomly generated fasta files in order to control for nucleotide variability and mitigate the impact of true biological diversity inherent to the studied taxa. Random fasta files were generated with the function fastaRandom .py, generating sequences of 1000 bases long. To recreate the different target and excluding files, the first sequence was randomly generated and the rest of the sequences contained a 1% mutation probability compared to the first sequence for the *target* file and 20% for the *excluding* file. Different file sizes were created containing from 10 up to 500,000 sequences (in a logarithmic series) for both the *target* and the *excluding* files. For simplicity, only *target* and *excluding* files containing the same number of sequences were compared. Quantitative performance analyses were run on an Ubuntu 24.04 laptop with 32 GB RAM and 16 physical cores (Intel Core i5‐1250P).

When it comes to run time, *findOligo*, *testOligo* and especially *testThorough* are the most expensive functions. They all showed an increase in computing time that was directly proportional to the number of sequences in the fasta files (Figure 4). The function *findOligo* showed a linear slope of 0.0015 s per sequence (of the *target* and *excluding* files) and no significant differences in the length of the oligonucleotide searched (Figure 4A). When mismatches were tested, the number of mismatches more significantly increased the running time than the size of the fasta file. The function *testOligo* showed slopes of 0.0081, 0.014 and 0.017 s per oligo compared against 1000 sequences when allowing 1, 2 and 3 mismatches respectively (Figure 4B). The slowest function of all is *testThorough*, showing slopes of 0.091, 1.14 and up to 8.48 s per oligo compared against 1000 sequences on average when allowing 1, 2 and 3 mismatches respectively (Figure 4C). In addition, *testThorough* showed significant differences in the length of the oligonucleotide tested (linear regression *p*‐value = 0.00494). Given these results, we recommend using the *testOligo* function to curate the oligonucleotides and apply a thorough test only in those highly relevant oligonucleotides with the function *testThorough*.

The most expensive functions in terms of memory usage are *findOligo* and *getHomolog*, consuming up to ~6 and ~18 GB respectively when using the largest *target* and *excluding* files (with 500,000 sequences each; data not shown). Unlike run time, the memory usage of *findOligo* depends mostly on the number of *k*‐mers in the given fasta file rather than the number of sequences, with up to 2 GB of memory difference in fasta files with different numbers of *k*‐mers but a similar number of sequences. While *findOligo* requires memory that is affordable in most modern desktop and laptop computers, *getHomolog* might require exceptional resources when considering very large datasets (> ~400 k sequences). The rest of the functions use a similar memory as the file size, being up to 1GB in the largest examples.

When using empirical datasets and analyses, as those used in the previous section (‘Recommendations based on a qualitative performance’; Table 1), run time and memory usage reach overall similar values to those obtained for random files for similar file sizes (Table S2). On average analyses took ~20 m 45 s and 3.2 GB of RAM usage (see Table S2 for details). The fastest and least computationally demanding analyses were achieved when using the database EukRibo (46,346 total sequences) with ~4 m 42 s on average and up to 1.5 GB of RAM. Using the database PR2 (221,085 sequences), analyses were completed on an average of ~10 m 45 s and up to 2.1 GB of RAM, with a maximum runtime of 45 m 12 s in specific cases. Lastly, using the database SILVA (510,495 sequences), analyses were completed on an average of ~44 m 49 s (up to 1 h 56 m 47 s in specific cases) and using up to 6.3 GB of memory.

## 6 Conclusion

With oligoN‐design, we attempted to generate a simple and versatile tool for the design of specific oligonucleotides that can be integrated into bioinformatic pipelines, requiring only 2 fasta files as input. Based on our experience in primer and probe design with a wide diversity of different approaches, here we streamlined and automatized the complementary expertise of the authors to facilitate the development of specific probes and primers. We will continue to improve the accuracy, speed and robustness of oligoN‐design in the future, adding new features and integrating further feedback from the community.

## Author Contributions

M.M.S. designed research, performed research, analysed data and wrote draft manuscript; M.W., C.B., R.M. and D.J.R. designed research; all authors contributed analytical tools and wrote final manuscript.

## Conflicts of Interest

The authors declare no conflicts of interest.

## Supporting information


**Table S1:** Qualitative performance of an unsupervised search on different marker genes (COI, 12S and 16S mitochondrial and ribosomal ITS) using comprehensive pan‐eukaryotic databases (eKOI, MIDORI2 and UNITE).
**Table S2:** Total run time and maximum RAM usage of the unsupervised pipeline run on Table 1 over the final curated datasets.
**Figure S1:** A detailed overview of the oligoN‐design main functions.

## Data Availability

The authors have nothing to report.
