# Supplementary material for: OligoN‐Design: A Simple and Versatile Tool to Design Specific Probes and Primers From Large Heterogeneous Datasets
Source: Mol Ecol Resour. 2026 Apr 12;26(3):e70140. doi: 10.1111/1755-0998.70140 (PMC13071497; doi:10.1111/1755-0998.70140)
Supplement: Supplementary file 1 — Table S1: Qualitative performance of an unsupervised search on different marker genes (COI, 12S and 16S mitochondrial and ribosomal ITS) using comprehensive pan‐eukaryotic databases (eKOI, MIDORI2 and UNITE). Table S2: Total run time and maximum RAM usage of the unsupervised pipeline run on Table 1 over the final curated datasets. Figure S1: A detailed overview of the oligoN‐design main functions. [file MEN-26-e70140-s001.pdf]

MOLECULAR ECOLOGY RESOURCES

Supplemental Information for:

OligoN-design: A simple and versatile tool to design specific probes and primers from large heterogeneous datasets

Miguel M. Sandin, Marie Walde, Nicolas Henry, Irene Forn, Nathalie Simon,
Cédric Berney, Ramon Massana, Daniel J. Richter

Table of Contents:

**Table S1**	Page 2
**Table S2**	Page 3
**Figure S1**	Page 4

Table S1. Qualitative performance of an unsupervised search on different marker genes (COI, 12S and 16S mitochondrial, and ribosomal ITS) using comprehensive pan-eukaryotic databases (eKOI, MIDORI2, and UNITE) showing the number of sequences in the target file (**N**), the average genetic diversity (measured as the average similarity identity by applying the '--allpairs_global' from VSEARCH) of the target file (**S**) whether the given probe was found (✔) or not (✘) (**F**), and the average length of all oligonucleotides found (**L**). 'NA' represents 'Not Applicable' (i.e., lack of oligonucleotides found or sequences too divergent to compute similarity), 'sd' represents 'standard deviation' of the given average.

Database	Group	N	S	F	L
eKOI	Bangiophyceae	30	75.7 sd: 8.18	✘	NA
	Chlorophyceae	84	66.0 sd: 9.22	✘	NA
	Dinoflagellata	251	87.9 sd: 7.75	✔	10.1 sd: 1.6
	Embryophyceae	216	87.0 sd: 6.23	✔	9.83 sd: 1.47
	Foraminifera	38	85.8 sd: 8.67	✔	11.8 sd: 2.71
	Glaucophyta	10	78.2 sd: 6.43	✔	9.5 sd: 2.12
	Metazoa	10993	64.7 sd: 5.8	✘	NA
MIDORI2-12S	Apicomplexa	156	55 sd: 16.4	✘	NA
	Cercozoa	7	60.5 sd: 12.4	✔	9.84 sd: 3.22
	Chlorophyta	374	49.2 sd: 7.36	✘	NA
	Ctenophora	20	54.9 sd: 6.54	✔	12
	Endomyxa	2	90.4	✔	27.5 sd: 5.86
	Haptophyta	54	58.0 sd: 13.5	✔	9.17 sd: 1.17
	Telonemia	2	86.8	✔	27.4 sd: 6.13
	Tubulinea	5	50.7 sd: 0.1	✔	8.76 sd: 2.96
MIDORI2-16S	Apicomplexa	436	51.5 sd: 13.5	✘	NA
	Cercozoa	13	50.6 sd: 11.7	✔	8
	Chlorophyta	726	47.2 sd: 6.53	✘	NA
	Ctenophora	28	58.7 sd: 7.49	✔	12 sd: 0
	Endomyxa	6	48.2 sd: 9.46	✘	NA
	Haptophyta	56	56.8 sd: 14.6	✔	9.18 sd: 1.92
	Telonemia	4	NA	✔	7.95 sd: 0.7
	Tubulinea	3	NA	✔	8.24 sd: 1.1
UNITE	Bacillariophyceae	269	58.5 sd: 8.45	✔	25.6 sd: 7.78
	Colponemidia	16	72.5 sd: 11.0	✔	13.9 sd: 9.25
	Copepoda	97	61.5 sd: 11.4	✔	14.4 sd: 5.67
	Dictyochophyceae	5	96.0 sd: 0.84	✔	28.6 sd: 4.56
	Endomyxa	199	68.9 sd: 10.9	✔	24.6 sd: 7.65
	Glaucophyta	2	62.9	✔	27.3 sd: 6.31
	Polycystinida	8	76.0 sd: 15.7	✔	24.6 sd: 8.8
	Saccharomycetes	1930	60.2 sd: 6.52	✘	NA

Table S2. Total run time and maximum RAM usage of the unsupervised pipeline run on Table 1 over the final curated datasets. Run time is given in human readable format (hours, minutes and seconds: h, m, s, respectively) and in seconds. RAM usage is given in gigabytes (GB, for human readability) and kilobytes (KB).

Database	Target	Time	seconds	RAM (GB)	RAM (KB)
EukRibo	Eukaryota	NA	NA	NA	NA
	Haptophyta	1m 16s	76	1.6	1678444
	MAST-01B	5m 44s	344	1.5	1616460
	MAST-12C	3m 46s	226	1.5	1616016
	Pelagophyceae	3m 10s	190	1.5	1615244
	MAST-07	2m 42s	162	1.5	1616160
	Minorisa minuta	13m 20s	800	1.5	1615584
	Novel-Clade-2	2m 55s	175	1.5	1615900
PR2	Eukaryota	1m 14	74	1.7	1880432
	Haptophyta	4m 40s	280	2.1	2222136
	MAST-01B	4m 57s	297	2.0	2095160
	MAST-12C	10m 05s	605	2.0	2098732
	Pelagophyceae	9m 26s	566	2.0	2098048
	MAST-07	7m 12s	432	2.0	2098508
	Minorisa minuta	45m 12s	2712	2.0	2098900
	Novel-Clade-2	3m 19s	199	2.0	2097428
SILVA	Eukaryota	10m 56s	656	3.7	3888000
	Haptophyta	14m 42s	882	6.3	6630540
	MAST-01B	15m 00s	900	6.3	6631560
	MAST-12C	34m 51s	2091	6.3	6631920
	Pelagophyceae	43m 36s	2616	6.3	6631944
	MAST-07	49m 07s	2947	6.3	6631748
	Minorisa minuta	1h 56m 47s	7007	6.3	6631552
	Novel-Clade-2	1h 13m 38s	4418	6.3	6631752

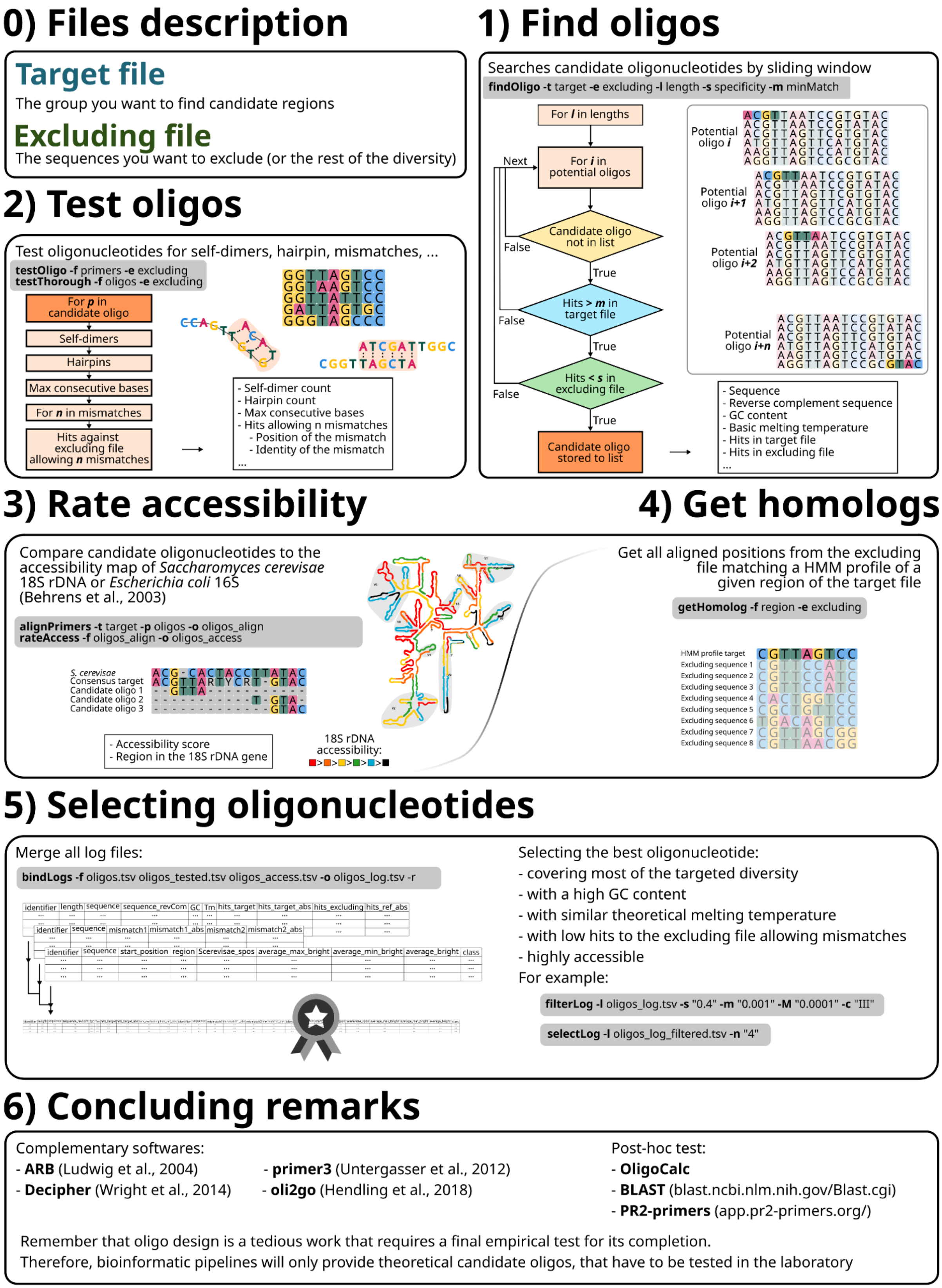

Figure S1. A detailed overview of the oligoN-design main functions.
